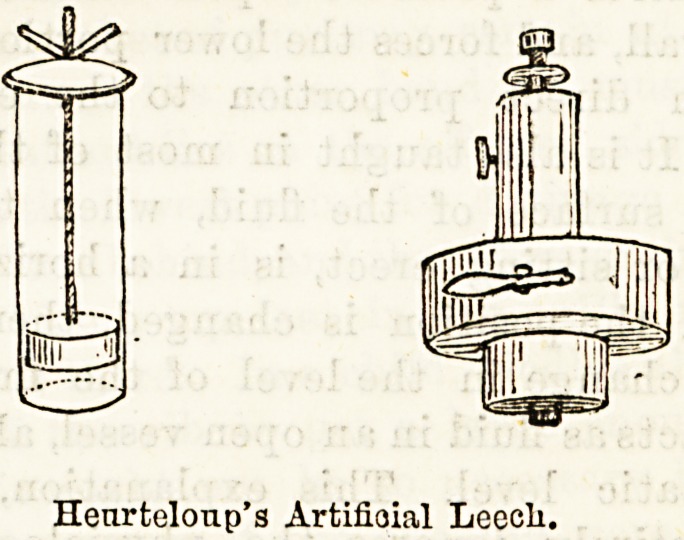# London Hospital—Treatment of Acute Iritis

**Published:** 1894-05-12

**Authors:** 


### LONDON HOSPITAL.

### Treatment of Acute Iritis.

In the treatment of acute iritis it is of importance to recognise from the first if there is any constitutional condition, either causing or predisposing to the attack; the constitutional conditions that most influence the course of the disease being rheumatism, syphilis, and gout. A few cases are associated with other conditions as diabetes, but a very considerable proportion of cases are idiopathic, no cause for their arising being discoverable. Apart from all the foregoing classes stands traumatic iritis, caused by some direct injury or wound of the iris. The treatment of iritis is necessarily local and constitutional; early and energetic local treatment being absolutely necessary to obtain a good result. The iris becoming inflamed and covered with plastic lymph is often glued down, and perhaps permanently fixed, to the capsule of the lens which lies just beneath it. If this unfortunate occurrence takes place while the pupillary aperture is small, serious impairment of vision may be the result; for not only may the iris be bound down and unable to dilate and contract in concurrence with the needs of the rest of the visual apparatus in accommodation and reaction of light, but the exudation of lymph may extend over the whole of the pupillary opening, forming a deposit on the front of the lens, preventing any distinct vision or leaving only perception of light.

Our object then is to obtain as great a dilatation of the iris as is possible early in the attack, for the double reason that when the pupil is dilated the fibres of the iris being necessarily squeezed together, less blood can be present in the muscle and less exudation take place, what exudation of lymph is present being less likely to spread over the lens capsule, leaving deposits of exudation on its surface.

Atropine is the drug most used for this purpose, and is suitable for the greater number of cases of acute iritis. Several methods of applying it are used, the commonest is a watery solution of four grains to the ounce of distilled water; this is dropped into the eye with a small glass pipette, which is simply a piece of glass tubing drawn out to a point, and having at the other end an unperforated indiarubber nipple; or one of the many forms of drop bottles may be used, all of which are very much more expensive and difficult to clean than the pipette, which is so cheap that it can be frequently renewed.

The solution is applied every three or four hours till the pupil is fully dilated, then three or four times in the course of the twenty-four hours, to keep it in this condition as long as may be required. Occasionally we use a much stronger solution, of ten grains to the ounce, when iritis has already been in existence for some time and adhesions have taken place, powerful dilatation of the iris being necessary to break them down. In some, children especially, there is a certain amount of difficulty in introducing drops, the eyelids being kept firmly closed, so that the drops are squeezed out as soon as put in, and the subsequent flow of tears effectually washes out the remainder of the atropine solution. The difficulty is met by substituting for the watery solution an ointment, made with atropine of the same strength, four to ten grains to the ounce of vaseline. This is most easily and safely applied with a small camel's hair brush, which is dipped in the ointment, the lower lid drawn down so as to evert it somewhat, the brush placed inside the lid and parallel to its long axis, and then the lids allowed to close on the brush. As it is withdrawn at the outer angle of the eye, the firm closure of the lids, which in children almost involuntarily takes place, effectually squeezes the ointment off the brush, It quickly dissolves in the eye, and is as a rule, a more certain application than a solution. Iodoform is often added to the ointment, especially when the case is one where there is some wound or abrasion of the cornea or coats of the eye, as in traumatic iritis with a wound in the cornea or ciliary region. For this purpose the iodoform must be of the precipitated, not the crystalline variety, which unless ground and sifted, is in too large scales for comfortable use in ophthalmic cases. Cocain is occasionally added to both the solution of atropine and the ointment; it is thought to increase the dilating power of the atropine, as well as easing pain, being used of varying strengths, four to twenty grains to the ounce. Every now and then a case is found where atropine is not tolerated, its use causing great swelling and redness of the lids and conjunctiva, with photophobia, and even some of the constitutional symptoms of atropine poisoning, dryness of the throat, quick pulse, and even delirium. In these cases it is advisable to substitute duboisin in a half per cent. solution, or homatropine; the former is the most efficacious, but has to be used with caution, as serious symptoms have followed its use. As a rule, a single drop of the solution is enough to apply at any one time, and the same may be said with regard to atropine, or any other solution. If more than a couple of drops are applied the surplus will run out again as soon as the lids close, the capacity of the lids for retaining fluids being extremely small. In some people slight dryness of the throat follows the use of atropine drops, and has been supposed to be due to some of the solution passing down the nasal duct to the throat and getting absorbed, giving rise to the constitutional symptoms. It is often stopped by keeping a finger pressed on the lachrymal duct for a minute after the application of the drops, to allow absorption to take place into the eye. When present it is relieved by small doses of pilocarpine, or gargling the throat with strong coffee, using cold or with ice.

Every now and then a pupil will not dilate with atropine or other mydriatic, even when there are few or no adhesions to prevent it. This most often occurs in rheumatic cases where there is severe pain; the atropine acts as soon as the pain has been relieved by bleeding, which is far and away the quickest and most efficacious way of relieving the pain of iritis. Bleeding may be performed either by means of leeches to the

Heurteloup's Artificial Leech.

temple, from six to twelve being used at a time, or the artificial leech is used, Heurteloup's being the most useful. This consists of a hollow steel cylinder rotated by a spring, the cylinder can be set by means of a screw to cut to any depth requisite, then blood is obtained from the cut by means of glass cylinders having tightly-fitting pistons, which when gradually screwed up produce a vacuum. Blood is obtained more quickly than by leeches, and for this reason this method is preferred by some. From two to eight ounces of blood can be rapidly extracted, and when bleeding is necessary relief is more complete and permanent if four to six ounces are taken away than a smaller quantity. Next to bleeding for the relief of pain comes the application of heat, which may be either dry or moist, very often the two forms alternated being found the most comfortable.

The eye is well bathed with hot water or boracic solution, then some cotton wool, preferably the non-absorbent form, wrung out of very hot water, poppy-head decoction, or weak watery solution of extract of belladonna is applied over the affected eye, covered with a piece of waterproof and cotton-wool, the whole being kept in place by a bandage. Dry heat is used by applying a large piece of gamgee or cotton wool, heated as hot as can be tolerated in front of the fire or on a hot-water tin, over the eye, temple, and forehead on the affected side, covering it over with some more wool and bandaging on. In all cases rest of both eyes is essential; even if one eye only is affected both must be shaded from the light, and the sound one used as little as possible, or not at all. When fomentations are not being used, either a large shade is kept over both eyes, the lower margin of the shade reaching as low as the tip of the nose, or dark goggles are worn. Cold is only applicable to iritis following injury, and even then is most useful in the first twenty-four to forty-eight hours. It is best applied by keeping the patient in bed, placing over the affected eye a piece of lint folded two thick, with a small piece of ice between the folds. The lint should come up over the forehead, when it can be kept in place by a single turn of bandage round the head, while the part over the eye is exposed to the air.

The constitutional treatment is directed to any of the conditions associated with the disease that may be present. Where there is a history of specific disease or signs of that condition are present, mercury, usually as the grey powder, with the compound ipecacuanha powder is given in small doses, but frequently. In those cases believed to be rheumatic, salicylate of soda or salicine is used, with in some cases iodide of potash; this latter drug being found useful in all forms of iritis. When gout is present, alkalies, especially the citrate and bicarbonate of potash, are given with iodide of potash, soda, or ammonia. Colchicum is given if there is an acute outbreak of gout, but is found also to sometimes relieve the pain of the iritis though no acute manifestation of gout is present. For the relief of pain antipyrin and phenacetin are sometimes used, but no drug is so effectual as morphia given hypodermically or by mouth. Care is taken in all cases to keep the intestinal tract in good condition, a sharp purge being administered at the beginning of the attack, followed up by occasional salines. The diet is regulated by the constitutional condition of the patient, an anæmic broken down subject requiring a more liberal dietary than a plethoric gouty individual, but while the attack is acute the dietary is kept somewhat restricted. Alcohol is scarcely ever required, and in most if not all cases, is positively harmful, even in those accustomed to its use.

## Figures and Tables

**Figure f1:**